# Risk Assessment of African Swine Fever Virus Exposure to *Sus scrofa* in Japan Via Pork Products Brought in Air Passengers’ Luggage

**DOI:** 10.3390/pathogens9040302

**Published:** 2020-04-20

**Authors:** Satoshi Ito, Jaime Bosch, Cristina Jurado, José Manuel Sánchez-Vizcaíno, Norikazu Isoda

**Affiliations:** 1 Research Center for Zoonosis Control, Hokkaido University, Sapporo 001-0020, Japan; isoda@czc.hokudai.ac.jp; 2 VISAVET Center and Animal Health Department, University Complutense of Madrid, 28040 Madrid, Spain; jbosch@ucm.es (J.B.); cjdiaz@ucm.es (C.J.); jmvizcaino@ucm.es (J.M.S.-V.); 3 Global Station for Zoonosis Control, Global Institute for Collaborative Research and Education (GI-CoRE), Hokkaido University, Sapporo 001-0020, Japan

**Keywords:** African swine fever, biosecurity, exposure assessment, import risk, risk analysis, wild boar

## Abstract

In recent years, African swine fever (ASF) has become prevalent in many areas, including Asia. The repeated detection of the ASF virus (ASFV) genome in pork products brought in air passenger’s luggage (PPAP) was also reported from Japanese airports. In the present study, the risk of ASFV exposure to susceptible hosts in Japan via three different pathways was assessed. Two quantitative stochastic risk assessment models were built to estimate the annual probability of ASFV exposure to domestic pigs, which could be attributed to foreign job trainees or foreign tourists. A semi-quantitative stochastic model was built to assess the risk of ASFV exposure to wild boar caused by foreign tourists. The overall mean annual probability of ASFV exposure to domestic pigs via PPAP carried by foreign job trainees was 0.169 [95% confidence interval (CI): 0.000–0.600], whereas that by foreign tourists was 0.050 [95% CI: 0.000–0.214], corresponding to approximately one introduction every 5.9 and 20 years, respectively. The risk of ASFV exposure to domestic pigs was dispersed over the country, whereas that of wild boar was generally higher in the western part of Japan, indicating that the characteristics of the potential ASF risk in each prefecture were varied.

## 1. Introduction

African swine fever (ASF) is a highly haemorrhagic and devastating disease in swine caused by ASF virus (ASFV). Susceptible hosts among pig species, soft ticks, and materials contaminated with ASFV play essential roles in the transmission of the disease [1]. In addition to direct contact between these hosts, indirect contact with materials contaminated with ASFV, such as swill feeding, is one of the crucial causes of ASF outbreaks, which may occur even in a distant place [2]. Detection and culling of infected animals or those suspected of infection and restriction of animal movements are the fundamental countermeasures for the control of contagious diseases, including ASF. The illegal trade in animal or pork products and the movement of ASF-affected wild boar play important roles in disease transmission to ASF-free territories [3]. None of the effective vaccines against ASFV have been commercialised to date [4], despite the high mortality rate among affected animals, and therefore control measures against ASF rely heavily on preventing the introduction of the disease into ASF-free territories by means of strict border controls. 

After ASF was introduced into Georgia in 2007, the disease immediately spread to the Caucasian region and then Eastern European territories mainly by the movement of wild boar or suspected illegal animal transportation [3]. In August 2018, the first ASF outbreak was reported in China. The results of a partial sequence of the ASFV p72 gene indicated that the outbreak isolate, ASFV-SY18, belonged to the genotype II group and had an identical nucleotide sequence to the Georgian (Georgia 2007/1), Russian (Krasnodar 2012 and Irkutsk 2017), and Estonian (Estonia 2014) isolates [5], meaning that ASF was potentially transmitted from those European territories where the disease was present. Since then, ASF outbreaks have been confirmed in a total of 11 territories in the Asian region including Cambodia, China, North Korea, Hong Kong, Indonesia, Laos, Myanmar, the Philippines, South Korea, Timor-Leste, and Vietnam [6].

Japan had remained free from several contagious animal diseases through the implementation of strict border controls at ports of entry. However, the recent introduction of transboundary animal diseases such as Foot-and-mouth disease (FMD) in 2010 and Classical swine fever (CSF) in 2018 indicates that contagious viruses can be introduced from infected areas [7,8]. As cases of CSF have continued to be notified despite vigorous efforts at disease control [9], it could be assumed that the introduction of ASFV in Japan will cause a similar, or perhaps even worse, scenario since no vaccines are available for ASF. 

In a previous study, the probability of ASFV introduction into Japan via pork products brought in air passenger’s luggage (PPAP) was evaluated [10]. The detection of ASFV genome in PPAPs confiscated at Japanese airports has been continually reported since the first ASF outbreak was notified in China [11]. According to a report from the Japan Animal Quarantine Service (JAQS), by the end of November 2019, the ASFV genome had been detected in a total of 83 confiscated PPAPs. ASFV was isolated from two of them, which might have been infective if susceptible hosts fed on them [11]. Considering this, there would be a chance of ASFV being introduced into Japan via PPAP and transmitted to susceptible hosts. 

The aim of the present study is to evaluate the probability of ASFV exposure to susceptible animals in Japan via PPAP by a combination of quantitative and semi-quantitative methods. Since legal pork products were not assumed to be contaminated with ASFV [10], we focused only on the risk of illegal PPAP. The obtained results could be useful for improving control measures to reduce the potential risk of exposure of ASFV to susceptible hosts, including domestic pigs and wild boars. 

## 2. Results

### 2.1. Risk of ASFV Exposure to Domestic Pigs Posed by Foreign Job Trainees

The overall mean annual probability of domestic pig exposure to ASFV via PPAP by foreign job trainees was estimated to be 0.169 (95% confidence interval (CI); 0.000–0.600), which approximately corresponds to one introduction every 5.9 years.

In terms of the highest risk territory, the top five highest risk territories were in Asia (Table 1). Vietnam was the highest risk territory, followed by the Philippines, Cambodia, China, and Mongolia.

In terms of the destination prefecture, the top three highest risk prefectures were located in the Kanto region, which is located in the eastern part of the main island of Japan, namely Chiba prefecture, Ibaraki prefecture, and Saitama prefecture. Results on the top ten highest risk prefectures are described in Table 2. 

### 2.2. Risk of ASFV Exposure to Domestic Pigs Posed by Foreign Tourists

The overall mean annual probability of domestic pig exposure to ASFV via PPAP by foreign job trainees was estimated to be 0.050 (95% CI; 0.000–0.214), which approximately corresponds to one introduction every 20 years.

In terms of origin territory, the top five territories with the highest risk were territories in Asia and Europe (Table 3). Vietnam was the highest risk territory, followed by the Philippines, China, the Russian Federation, and Cambodia. 

In terms of the destination prefecture, the risk was dispersed around the whole of Japan. The Chiba prefecture was at the highest risk, followed by the Aichi prefecture, Nagasaki prefecture, and Oita prefecture. Results on the top ten highest risk prefectures are described in Table 4.

### 2.3. Mean Annual Risk of Exposure of ASFV to Domestic Pigs 

**Figure 1:**
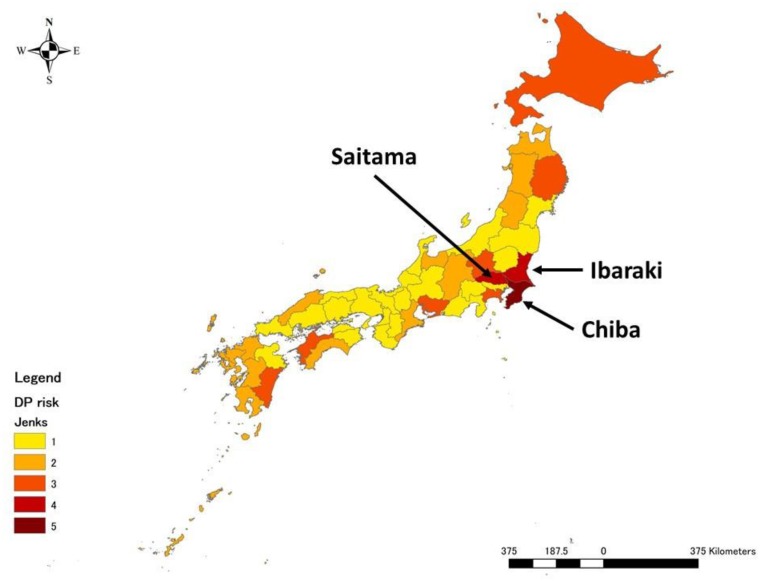
Mean annual risk of African Swine Fever Virus (ASFV) exposure to domestic pig via pork products brought in air passengers’ luggage at the prefecture level. The graduated colour map represents the relative risk from the highest (darker) to the lowest (lighter).

The risk of ASFV exposure to domestic pigs posed by foreign job trainees and foreign tourists were summed up to calculate the total risk to domestic pig, and then a risk score from 1 to 5 was assigned based on Jenks natural breaks (Figure 1). The obtained results showed that prefectures with high risks were located in the Kanto region. Chiba prefecture was found to be the prefecture at the highest risk (5), followed by Ibaraki (4) and Saitama (4) prefectures.

### 2.4. Risk of ASFV Exposure to Wild Boar

In terms of the origin territory, the final output indicated that the highest risk of wild boar exposure to ASFV via PPAP came from Vietnam (5), followed by Cambodia (4), the Philippines (4), the Russian Federation (4), China (3), Ethiopia (3), and South Korea (3) (Table 5). It is understandable that territories in the Asian region should present the highest risk of exposure to ASFV for wild boar in Japan, given the higher numbers of visitors and ASF status in the origin territory, although the Russian Federation and Ethiopia were ranked in the top seven highest risk territories. All territories not given in Table 5 were assigned a score of 1.

**Figure 2:**
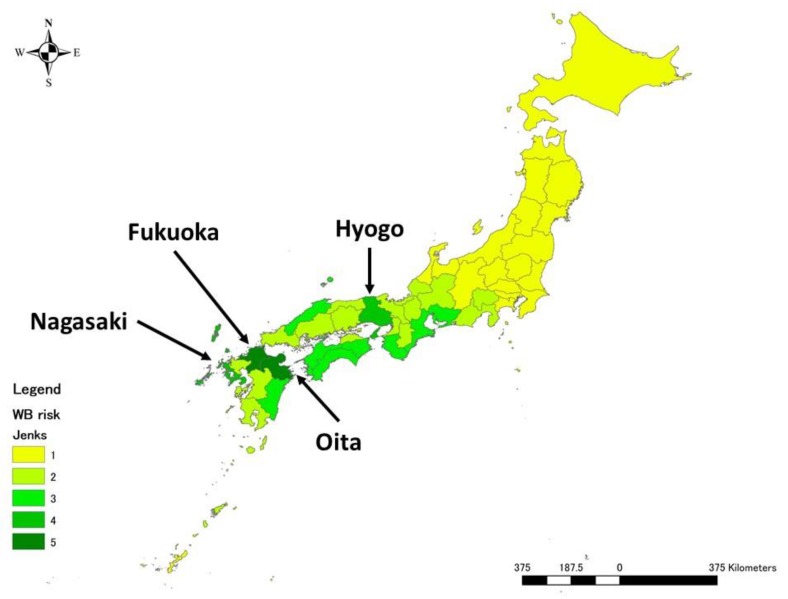
Mean annual risk of ASFV exposure to wild boar via pork products brought in air passengers’ luggage at the prefecture level. The graduated colour map represents the relative risk from the highest (darker) to the lowest (lighter).

In terms of destination prefectures, prefectures in the Kyushu region, which is an island located in the western part of Japan, were categorised as high risk (Figure 2). The two prefectures of Fukuoka and Oita in Kyushu had the highest risk (5), followed by Hyogo (4), Nagasaki (4). For the other prefectures, eight prefectures were assigned the score of 3, 16 prefectures scored 2, and the remaining 19 prefectures scored 1. Overall, the western part of Japan was likely to be at high risk of wild boar exposure to ASFV, whereas the eastern and northern parts of Japan were likely to be at low risk of it. 

### 2.5. Sensitivity Analysis

**Figure 3:**
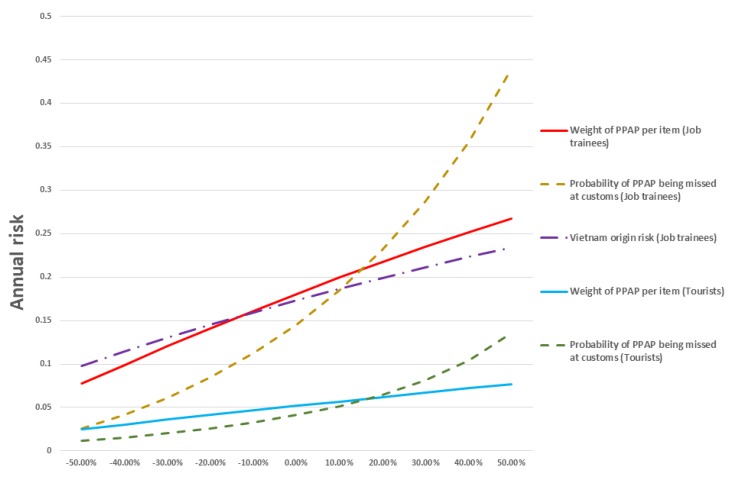
Results of the advanced sensitivity analysis for the risk of ASFV exposure to domestic pigs in Japan via pork products brought in air passengers’ luggage. The horizontal axis shows the percentage change of the selected input parameters against the annual risk in the vertical axis.

The Spearman correlation coefficients indicated that the weight of PPAP coming into Japan, the probability of PPAP being missed at customs, and the probability of ASF infection in Vietnam were highly-correlated input parameters in the model of domestic pigs exposed to PPAP by foreign job trainees. In the model of domestic pigs exposed to PPAP by foreign tourists, the weight of PPAP coming into Japan and the probability of PPAP being missed at customs were highly correlated with the output parameters used. The advanced sensitivity analysis revealed that the probability of PPAP being missed at customs was the most influential parameter among the three parameters, followed by the weight of PPAP coming into Japan in both models (Figure 3). The results of the jackknife sensitivity analysis showed that the probability of wild boar suitable habitat was the most influential estimator, explaining 57.9% of the semi-quantitative assessment results. The wild boar distribution explained 40.0% of the assessment results, followed by the territory of origin risk (24.6%), tourist-related risk (24.6%), and risk of direct or indirect contact between tourists and wild boars (19.7%).

## 3. Discussion

Several studies have assessed the risk of ASF introduction through various pathways [3,12,13,14,15,16]. However, epidemic ASF conditions require exposure risk assessments since the detection of ASFV-contaminated items has been reported in several territories [17,18,19,20]. Although the higher risk of disease in airports in Japan and origin territories were identified in the previous study [10], taking into consideration that the number of ASF outbreaks and affected areas in Asia have been continually increasing, it was necessary to conduct this in-depth study to protect Japan from ASF outbreaks. A total of 83 confiscations of PPAPs contaminated with ASFV were reported in Japan’s 12 airports up to the end of November 2019 [11], demonstrating that contagious pathogens have already reached the border of Japan and have the potential to penetrate inside the nation. Given this situation, JAQS has strengthened border controls by imposing penalty charges, increasing the number of detection dogs, and increasing the number of flights to be investigated [21]. However, it is difficult to investigate PPAPs of all passengers arriving in Japan, so the risk of ASFV being introduced into Japan is not negligible. Therefore, the prevention of ASF outbreaks depends on the effectiveness of preventive measures by competent authorities. Actually, under Japanese regulations, each of the prefectures is responsible for containing contagious animal diseases. Risk assessment at the prefecture level should help to identify the necessary measures for prevention of and reaction to ASF outbreaks once they occur. 

In the present study, the risk of exposure to ASFV via PPAP among susceptible animals in the prefectures of Japan was analysed in relation to three pathways. This was the first study to quantify the risk of foreign job trainees, even though the accessible data was limited. The obtained results indicated that foreign job trainees from two territories present more than 99% of the total risk; the highest risk territory, Vietnam, accounted for 97% of the total risk, followed by the Philippines (2.5%). These findings can be attributed to several factors—the average weight (kg) of PPAP not confiscated at quarantine per person per territory, the number of job trainees working in Japan per territory per prefecture, and the probability of PPAP contaminated with ASFV arriving from these territories. The availability and quality of data are always major limitations for any risk assessment. In the current analysis, the average weight of PPAP per person was applied instead of considering the risk distribution, though it is unlikely that all passengers bring pork products when travelling. The proportion of passengers with PPAP and its weight (kg) per territory of origin is required for further assessment. According to the 2017 Japan Pork Producers Association (JPPA) report, more than 36% of foreign job trainees come from Vietnam, followed by China (21.9%) and the Philippines (11.7%). Although job trainees from China were the second-highest in number, the overall risk derived from Chinese job trainees was lower than those from the Philippines, and Cambodia in the present study. Chinese visitors had the highest number of confiscated pork products, but the average weight of the PPAPs per person from China was lower than that from Vietnam, the Philippines and Cambodia. In addition to these factors, the current level of ASF infection in each territory also contributed to the results. In China, the first ASF outbreak was confirmed in August 2018; since then, 163 outbreaks had been notified, and about 1.2 million pigs had been culled as of December 5, 2019 [6]. However, an enormous number of ASF outbreaks have been reported in Vietnam as well. Since the first ASF outbreak was confirmed on February 19, 2019, almost 8500 outbreaks had been detected, and more than 5.9 million pigs had been culled in Vietnam by November 10, 2019 [6,22].

In terms of the prefectures at risk, all three prefectures with the highest risk were located in the Kanto region. According to the JPPA report, about 35% of farms in the Kanto region accept foreign job trainees, whereas, in the Kyushu region, where the largest number of pigs are reared, only 3.6% of the farms accept foreign job trainees. As not all farmers participated in the survey, the risk rank would change if all information about foreign job trainees was available. 

The risk of domestic pig exposure to ASFV via PPAP carried by foreign tourists was also evaluated in the present study. The obtained results indicated that the risk of the origin territory is more widely dispersed compared with the results of the scenario for foreign job trainees. Although 95% of the risks were derived from Vietnam (81%) and the Philippines (14%), European territories, including the Russian Federation, Poland, Germany, and Italy were listed in the top ten highest risk territories. The current ASF epidemic in Asia has given Japan warning of the considerable threat of ASF from the Asian region; however, the obtained results indicate that the risk of ASF transmission from Europe should not be ignored.

It was demonstrated that 90% of the total risk of domestic pig exposures to ASFV caused by foreign tourists was concentrated in the top ten highest risk prefectures; more than 50% of the entire risk was concentrated in the top three highest risk prefectures of Chiba (23%), Aichi (18%) and Nagasaki (10%). Tourists coming to Japan to see their friends or family may deliver PPAP to pig farmers. Therefore, the risk is likely to be influenced by the number of tourists coming to see friends or family and the proportion of pig farmers per total population in each prefecture.

In the present study, the annual risk of domestic pig exposures to ASFV via PPAP carried by foreign job trainees was 3.4 times higher than that presented by foreign tourists. It is generally held that the risk attributed to foreign job trainees is higher than that attributed to tourists. Our current approach demonstrated the potential risk posed by foreign job trainees. According to our assumptions, the risk of ASFV introduction onto farms should be influenced by the level of farm biosecurity and workers’ behaviour. Even if employees are educated in farm biosecurity by employers, the virus could be introduced into farms if workers do not follow instructions. 

The management of farm biosecurity is the most basic but most important factor for the prevention of the disease. We adjusted the questionnaire survey regarding farm biosecurity to address more specifically the risk of introduction of ASFV in order to clarify the importance of each preventive measure by gathering expert opinions. In the questionnaire, some of the questions were consistently scored as important by our experts, whereas opinions varied with regards to other questions such as the recording of visitors’ details. Most of the experts agreed with the importance of the disinfection of vehicles and countermeasures against the invasion of wild animals, whereas there were various opinions about human movements on farms. The obtained results will be useful for reviewing farm biosecurity in each prefecture. Unfortunately, as information for several variables was limited, according to the principle of maximum risk, we considered the worst-case scenarios for these; all leftover meat was fed to pigs, and all tourists visiting farmers delivered their PPAPs to those farmers. Although these variables were not indicated as influential parameters in the sensitivity analysis, further assessment is required to more accurately estimate these risks.

The risk of wild boar exposure to ASFV via PPAP was also evaluated as one of the scenarios in the current study. It is generally held that continuous ASF outbreaks in Europe can be attributed to the movement of affected wild boar as well as human movements [1]. Taking into consideration the fact that wild boar played an essential role in the Japanese CSF outbreak in 2018 [9], it can be easily assumed that ASF could have also been widely prevalent in Japan through successful contact among wild boars. The obtained results showed that the risk of wild boar exposure to ASF tends to be clustered in the western part of Japan; prefectures with a higher risk are centred in the Kyushu region. These results might be attributed to the probability of the existence of suitable habitat areas for wild boar and the wild boar distribution as well as the high number of tourists potentially having contact with wild boar. As the largest number of domestic pigs are in the Kyushu region, tremendous damage to the swine industry may be caused if ASF becomes prevalent, as was the case in the FMD outbreak in Miyazaki in 2009 [7]. In terms of the risk presented by wild boar distribution, a total of five prefectures were assigned a score of 4 to 5, but none of the prefectures in the eastern part of Japan were included. In contrast to the western part of Japan, almost all prefectures in the eastern and northern parts of Japan were assigned a score of 1. 

A similar trend was observed in the probabilities relating to wild boar suitable areas and potential contact between tourists and wild boar. Although some prefectures in the eastern part of Japan were assigned relatively high scores according to some risk estimators, none of these prefectures was assigned a high score in other risk estimators, and therefore, the overall risk score of prefectures in the eastern part of Japan was 1. 

The first CSF outbreak for the last 26 years was reported in Gifu prefecture in 2018 [9]. Considering that levels of farm biosecurity and the movement of wild boars are critical factors for managing both diseases, it is logical to think that the areas affected by CSF could also be potentially high-risk areas of ASF introduction, although the origin of the two disease introductions would not be same. The results of sensitivity analysis for the risk of domestic pigs caused by foreign job trainees indicated that the number of foreign job trainees and their behaviour on farms could contribute to the risk of ASFV exposure to domestic pigs (Data are not shown). The results of the jackknife sensitivity analysis indicated that the probability of wild boar suitable habitat was the most influential risk estimator for the risk of wild boar. Taking into account that there are higher numbers of pig farms accepting foreign job trainees in Tokai region, which Gifu prefecture belongs to [23], and the Gifu prefecture has wild boar suitable habitat, the obtained results are reasonable since these are parts of common risk factors among ASF and CSF. However, the results do not indicate a high ASF risk for Gifu prefecture. Gifu might have a lower risk for ASF than CSF introduction because of different contact rates with ASF and CSF infected areas.

The results of this study indicate that the characteristics of the potential risk of ASFV introduction in each prefecture are varied; therefore, it is essential to understand the factors contributing to the risk of disease invasion to each prefecture. The obtained results could be useful for launching or improving effective control measures to prevent the introduction of ASFV and for reviewing the current risk reduction activities in the field.

## 4. Materials and Methods

### 4.1. Concept of the Risk Assessment Model

Two quantitative stochastic risk assessment models were built to estimate the annual probability of ASFV exposure to domestic pigs that could be attributed to foreign job trainees or foreign tourists. The risk of ASFV exposure to wild boars via illegal PPAP was assessed semi-quantitatively because there were some difficulties in terms of data accessibility. Following the previous study [10], air passengers coming to Japan with illegal PPAPs from 47 origin territories with commercial routes to Japan were regarded as potential contributors to the exposure of ASFV to susceptible hosts in each of the 47 prefectures of Japan. Flight information from August 2016 to October 2018 was obtained from the Ministry of Land Infrastructure Transport and Traffic (MLIT) database [24]. Datasets on confiscated pork products, some of which contained ASFV genes, were obtained from the JAQS database [11,25]. Information about ASF disease status and the number of susceptible pigs in each territory of origin was obtained from the OIE–WAHIS as of November 30, 2019 [26] and The Food and Agriculture Organization Corporate Statistical Database (FAOSTAT) 2018 [27], respectively. The risk model was developed using @RISK 7.6 (Palisade Corporation, Newfield, NY, USA) in Microsoft Excel (Microsoft, Redmond, WA, USA), and 10,000 iterations were run using the Latin hypercube sampling method.

### 4.2. Structure of the Quantitative Risk Assessment Model for Domestic Pigs

**Figure 4:**
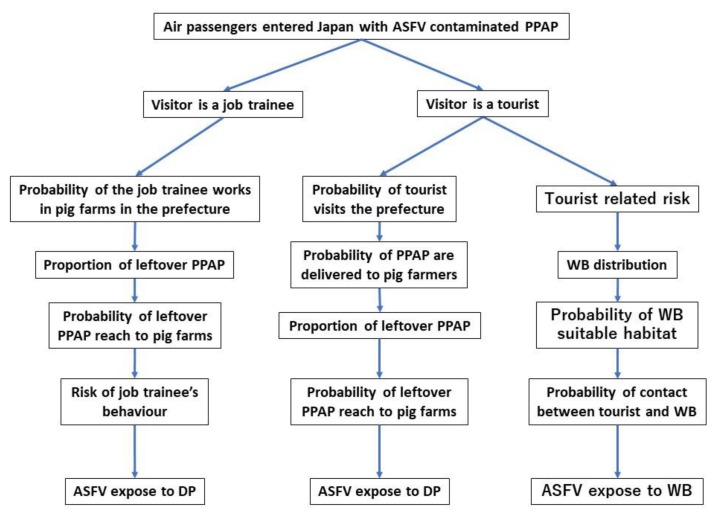
The flowchart shows the structure of the models to estimate the risk of ASFV exposure to susceptible animals in each prefecture of Japan via PPAP. Foreign job trainees and foreign tourists coming to Japan could have risk of ASFV exposure to domestic pigs (DP). Foreign tourists coming for sightseeing could have a risk of ASFV exposure to wild boars (WB).

Two quantitative stochastic models, one for foreign job trainees and the other for foreign tourists, were built to assess the annual probability of ASFV exposure to domestic pigs in the 47 prefectures of Japan via PPAP. In the present study, it is assumed that all domestic pigs on a farm have been exposed to ASFV once the virus has reached it. Each quantitative model followed a binomial process according to the formula below:

(1) $$P\left(x\geq 1\right)=1-\Pi \Pi {\left(1-P_{asf{\_}_{o}}\right)}^{V_{od}}{\;}^{\;}$$

where $V_{od}$ is the assumed weight (kg) of PPAP exposed to susceptible hosts in each prefecture (*d*) by visitors from each origin territory (o), $P_{asf{\_}_{o}}$ is the estimated probability of at least 1 kg of PPAP being contaminated with ASFV at each origin territory, and P(x ≥1) refers to the probability of ASFV being exposed to domestic pigs considering all the different visitors to Japan. Thus, the model consists of two main components—the quantity of PPAP to which domestic pig is exposed and the probability of PPAP being contaminated with ASFV. All input values, parameterisations, and references are presented in Table 6 and are described in the following sections. The outline of the scenarios is described in Figure 4. 

### 4.3. Weight of PPAPs Introduced into Japan (V_enter_vo_)

In the present study, PPAPs missed at the border by JAQS were regarded as the products that carried the risk of exposing susceptible animals to ASFV. The weight of PPAP (kg) not being detected by border controls per visitor per origin territory (*V*_enter*_vo*_) was calculated. The value of *V*_enter*_vo*_ was calculated by dividing the annual weight of PPAP (kg) being missed by border controls per territory (*V*_miss*_o*_) by the annual number of visitors coming to Japan from the given territory (*N*_all*_o*_). The value of *V*_miss*_o*_ was calculated by multiplying the probability of PPAP not being detected by border controls (*P*_miss_) by the weight of PPAP (kg) confiscated per origin territory (*V*_conf*_o*_). None of the data or expert opinions that would have allowed us to determine the value of *P*_miss_ were available; therefore, this was set as a triangular distribution that had minimum, most likely and maximum values of 0.2, 0.5, and 0.9, respectively, following the approach used in previous studies [10,14]. The value of *V*_conf*_o*_ was calculated by multiplying the annual number of PPAPs confiscated by border controls per territory (*N*_conf*_o*_) by the weight of PPAP (kg) per item (*V*_ppap_). The data on the annual number of PPAPs confiscated by border controls per territory was obtained from the JAQS database [25]. As the information on the weight of pork products confiscated at Japanese international airports, in which ASFV genes were detected for the period of October 2018 to September 2019 was available from the JAQS database, the value of *V*_ppap_ was set as a triangular distribution that had minimum, most likely and maximum values of 0.04, 1.1, and 9.7, respectively [11]. 

The value of *N*_all*_o*_ was estimated using data from the air passengers’ survey for the period from 2015 to 2017 conducted by the MLIT [29]. Although the survey includes information on the origin territories of tourists, their destination prefecture, and the purpose of their visits, the data was not covered for a whole year. Therefore, an annual number of tourists per territory was estimated using the formula below. The annual number of air passengers coming to Japan (*N*_all_) was available from the Statistics on Legal Migrants [28]:

(2) Nall_o=Nqall_o×(Nall/Nqall)

where Nqall_o and Nqall are the numbers of tourists coming to Japan per territory and in total, respectively, the number Nall_o was assumed to follow a normal distribution.

### 4.4. Number of Foreign Job Trainees Working on Pig Farms (N_jt_od_)

The adjusted annual number of foreign job trainees working on pig farms per origin territory per prefecture (*N*_jt*_od*_) was estimated using the 2017 pig farming survey of Japan (2017 JPPA report), conducted to investigate the overall condition of farm management [23]. Although the information on the number of foreign job trainees per territory was provided by farmers in the survey (*N*_qjt*_od*_), not all pig farmers in Japan participated in the survey. Therefore, the value of *N*_jt*_od*_ was estimated in the present study by the value of *N*_qjt*_od*_. 

First, a proportion of farms accepting foreign job trainees was calculated by dividing the obtained number of farms accepting foreign job trainees (*N_q_*_fjt*_d*_ or *N_q_*_fjt*_r*_) by the total number of farms participating in the survey (*N*_qf*_d*_ or *N*_qf*_r*_) at the prefecture level (*W*_fjt*_d*_) and the region level (*W*_fjt*_r*_), according to the 2017 JPPA report. These values were used to calculate the adjusted proportion of farms accepting foreign job trainees (Mod-*W*_fjt*_d*_). The value of Mod-*W*_fjt*_d*_ was set as a uniform distribution between *W*_fjt*_d*_ and *W*_fjt*_r*_. 

Second, the estimated number of farms accepting foreign job trainees per prefecture (*N*_fjt*_d*_) was calculated by multiplying the value of Mod-*W*_fjt*_d*_ by the number of pig farms per prefecture (*N*_f*_d*_) obtained from the 2017 Statistical Survey on Livestock [30]. The adjusted annual number of foreign job trainees working on pig farms per origin territory per prefecture (*N*_jt*_od*_) was finally calculated using the following formula:

(3) Njt_od=(Nfjt_d/Nqfjt_d)×Nqjt_od

### 4.5. Annual Weight of PPAPs (kg) Brought into Japan by Foreign Job Trainees (V_jt_od_)

The annual weight of PPAPs brought into Japan by foreign job trainees per origin territory per destination prefecture (V_jt*_od*_) was calculated by multiplying the value of *N*_jt*_od*_ by the value of *V*_enter*_vo*_. 

### 4.6. The Amount of Cumulative Exposure of PPAP (kg) to Domestic Pigs by Foreign Job Trainees (V_jexp_od_)

After calculating V_jt*_od*_, the amount of cumulative exposure of PPAP (kg) to domestic pigs by foreign job trainees (*V*_jexp_*od*_) was calculated as the product of the annual weight of PPAPs brought into Japan by foreign job trainees (*V*_jt_*od*_), the proportion of leftover PPAPs (*W*_left_), number of visit days to pig farms by foreign job trainees (*N*_vis_), and potential risk of leftover PPAP reaching pig farms (*P*_ent_*od*_). Each model input is described in the following sections.

### 4.7. The Proportion of Leftover PPAPs (W_left_)

In the present study, we assumed that leftover PPAP could reach pig farms and expose to susceptible animals. The data on the proportion of meat left over per family was used for estimating the proportion of leftover PPAPs [31]. The average proportion of meat leftover per family (*W*_left_) was set as 0.03.

### 4.8. Number of Visit Days to Pig Farms by Foreign Job Trainees (N_vis_)

Throughout the year, foreign job trainees have opportunities to visit farms. If biosecurity rules are not followed, foreign job trainees could carry and introduce potentially contaminated PPAP in domestic pig farms. In general, the number of working days is stipulated by the Labor Standard Law (LSL) [32]. According to the LSL, workers need to take at least one day off per week, indicating that there are approximately 313 days per year on which foreign job trainees could visit farms. We regarded that the number of days visiting pig farms can be interpreted as the number of potential opportunities that PPAPs expose to domestic pigs in the worst-case scenario. Hence, the value of *N*_vis_ was set as 313 in the present study. 

### 4.9. Potential Risk of Leftover PPAP Reaching Pig Farms (P_ent_od_)

The probability of PPAP being brought onto farms would be affected by farms’ biosecurity measures and workers’ behaviour. Farm biosecurity measures are usually established by employers, whereas employee behaviour is influenced by personal factors. According to these assumptions, the potential risk of leftover PPAP reaching pig farms (*P*_ent*_od*_) was calculated as the product of the level of farm biosecurity per prefecture (*P*_bs*_d*_) and the potential risks arising from workers’ behaviour (*P*_bhv*_o*_).

The value of *P*_bs*_d*_ was estimated by the 2016 JPPA report [33]. In the report, farmers replied to questions about the level of biosecurity on their farms with ‘yes’ (score of 1) or ‘no’ (score of 0), so the total score of farmers’ responses could be used to represent an index of farm biosecurity. Although the questionnaire was prepared to take into consideration animal hygiene standards, it was not designed to assess measures to control ASF specifically, but swine diseases more generally. To identify effective measures to prevent ASF, each question was weighted based on expert opinion. In the present study, we convened a total of 12 specialists with extensive field experience and knowledge of farm biosecurity and ASF. Six of them were invited from Spain, given that this territory experienced ASF outbreaks during past decades; the other six were from Japan, the territory serving as the research object. The 12 experts were asked to grade the importance of each question from zero (not important) to five (very important). The final weight of each question was determined using the median values of the expert responses. The degree of consensus among the experts was measured using the coefficient of variation, which is the rate of the standard deviation to the mean. The results from the experts’ opinions are shown in Table 7. 

The weighted total score of farm biosecurity per farm was calculated by summing up the farmer’s responses to the weighted score of each question and subsequently compared with theoretical maximum scores [e.g., the level of each farm biosecurity was scored from 0 (min.) to 1 (max.)]. The value of *P*_bs*_d*_ was then set as a triangular distribution that had minimum, most likely, and maximum values of the weighted total score of farm biosecurity per prefecture. 

The obtained results indicate that the levels of farm biosecurity were widely different among prefectures. The scores were distributed between the highest score of 0.73 and the lowest score of 0.20, with an average score of 0.49.

The risk of exposing pigs to ASFV could be influenced by the behaviour of employees on farms. In Japan, the production of eco-feed, which is made from recycled food waste, scraps, and leftover animal feed, has increased in recent years [37]. Exposure of PPAP to farm pigs through eco-feed could happen because employees, especially those with lower compliance, may not realise that eco-feed could contain PPAP. To quantify the potential risk of employee attitude, we calculated the proportion of farms using eco-feed (*W*_eco_), based on the 2017 JPPA report [23]. We considered that employee attitude, i.e., whether they feed swill or not, could depend on the lifestyle and culture of the country they belong to. As the quality of life can be associated with social compliance, and a low level of social compliance might cause illegal or unethical activities, a quality of life index per territory (*I*_life*_o*_) was used to quantify the potential risk per territory arising from workers’ behaviour (*P*_bhv*_o*_) [34]. The value of *I*_life*_o*_ was compared with that of Japan and then multiplied by the value of *W*_eco_ to calculate the value of *P*_bhv*_o*_. The quality of life index used was an estimation of the overall quality of life using an empirical formula that considered eight indices relating to human life [38].

### 4.10. Probability of PPAP Contamination with ASFV (P_asf_o_)

To estimate the probability that PPAP was contaminated with ASFV (*P*_asf*_o*_), the same approach as in a previous study was applied [10,14]. Briefly, a total of 47 origin territories that had direct or indirect flights to Japan were classified into three risk categories based on information about the level of ASF in each territory as at November 30, 2019 [39]: For territories in the ‘High’ risk category, the territory risk was set as a beta distribution of the potential number of non-reported ASF-infected domestic pigs in each territory per month and the estimated number of slaughtered domestic pigs in each territory per month. For territories in the ‘Medium’ or ‘Low’ risk categories, the territory risk was estimated by considering the probability of an outbreak occurring in the territory, the average size of an ASF outbreak, duration of the infection, probability of outbreaks being undetected and proportion of pigs to be slaughtered every month. For each of these categories, it was assumed that 1 kg of PPAP contaminated with ASFV indicated that at least one domestic pig was infected in the territory. Further detail of the risk calculation method is available from the previous study [10].

### 4.11. The Amount of Cumulative Exposure of PPAP (kg) to Domestic Pigs by Foreign Tourists (V_fexp_od_)

The amount of cumulative exposure of PPAP (kg) to domestic pigs by foreign tourists (*V*_fexp_*od*_) was calculated following a similar approach as that applied for calculating the risk of domestic pigs being exposed to PPAP by foreign job trainees. Each model input is described in the following sections.

### 4.12. Number of Foreigners as Tourists (N_frd_od_)

In the present study, tourists coming to Japan to see their friends or family were also assumed to have the potential to introduce ASF onto pig farms. As described above, the coverage of the survey of air passengers’ movements [29] was limited to a certain period, and therefore the annual number of tourists coming to Japan to see their friends or family (*N*_frd*_od*_) was estimated following the approach mentioned previously. The annual number of tourists per origin territory per destination prefecture was calculated according to the following formula:

(4) Nall_od=Nqall_od×(Nall / Nqall)

where *N*_qall_*od*_ is the total number of tourists coming to Japan per territory per prefecture during the period of the survey. The obtained value of *N*_all_*od*_ was assumed to follow a normal distribution.

The value of *N*_frd*_od*_ was calculated using the following formula:

(5) Nfrd_od=Nall_od×(Nqfrd_od/Nqall_od)

where *N*_qfrd*_od*_ is the number of tourists coming to Japan to see their friends or family per territory per destination prefecture during the period of the survey. The obtained value of *N*_frd_*od*_ was assumed to follow a normal distribution.

### 4.13. Probability of Tourists Providing PPAP to Pig Farmers (P_pass_d_)

In the present study, we assumed that tourists’ PPAPs could reach pig farms only when their friends or families were close to pig farms or pig farmers. We assumed that in the worst scenario, PPAPs were delivered to pig farmers directly. The probability of tourists providing PPAPs to pig farmers (*P*_pass*_d*_) was assumed to be the same as the probability of tourists meeting pig farmers in the present study. Therefore, the value of *P*_pass*_d*_ was calculated by dividing the number of pig farmers per prefecture (*N*_pf_*d*_) by the total population per prefecture (*N*_pop_*d*_). The number of pig farmers per prefecture was not available in the present study, so the value of *N*_pf_*d*_ was estimated based on the 2016 JPPA report and the 2017 Statistical Survey of Livestock [30]. Information on the number of employees and the size of the farm were provided in the 2016 JPPA report. The size of pig farms was classified into seven grades based on the animal census: 1–99, 100–299, 300–499, 500–999, 1000–1999, 2000–2999, >3000 pigs, according to the survey [30]. The average number of employees per farm size (*N*_emp_*s*_) was calculated using a uniform distribution that had minimum and maximum values per farm size [33]. The obtained value of *N*_emp_*s*_ was then multiplied by the number of pig farms per farm size per prefecture (*N*_f_*sd*_) to calculate the number of pig farmers per prefecture. It was also expected that PPAPs could be brought into pig farms after they were delivered by tourists to each of the farm members. To calculate the total number of potential recipients of PPAPs, the obtained value of *N*_pf_*d*_ was multiplied by the average number of family members in Japan (*N*_fami_) using the data set from the Outline of Health, Labour and Welfare Statistics [36]. Finally, the value of *P*_pass*_d*_ was calculated using the following formula:

(6) Ppass_d=(Npf_d×Nfami)/Npop_d

### 4.14. Annual Weight of PPAPs (kg) Delivered to Pig Farmers (V_pass_od_)

First, the annual number of tourists delivering PPAPs to pig farmers (*N*_pass*_od*_) was calculated by multiplying the value of *N*_frd*_od*_ by the value of *P*_pass*_d*_. The annual weight of PPAPs given to pig farmers (*V*_pass*_od*_) was then estimated as the product of the value of *N*_pass*_od*_ and the value of *V*_enter*_vo*_.

### 4.15. The Proportion of Leftover PPAP (W_left_)

The proportion of leftover PPAP, which was not consumed by human, was assumed to be the same as in the case of foreign job trainees. That is, the average proportion of meat leftover per family (*W*_left_) was set as 0.03.

### 4.16. Number of Days Farmers Visit Pig Farms (N_vis_)

Although no apparent restriction on working days was set for farmers in the LSL [32], it is likely that they have days off during the week. Therefore, the same methodology was also applied to calculate the potential risk of uneaten PPAP reaching pig farms (*P*_ent*_od*_) and the probability of PPAP being contaminated with ASFV. The value for the number of days that farmers visit pig farms (*N*_vis_) was the same as the one used in relation to foreign job trainees.

### 4.17. Annual Probability of Domestic Pigs’ Exposure to ASFV via PPAP (R_dp_od_)

Finally, the overall annual risk of domestic pig exposure to ASFV via PPAP (*R*_dp*_od*_) was calculated using the following formula:

(7) $$R{\mathrm{dp}}_{od}=P\mathrm{dp}-\mathrm{jt}\_od+P\mathrm{dp}-\mathrm{ft}\_od-P\mathrm{dp}-\mathrm{jt}\_od\times P\mathrm{dp}-\mathrm{ft}\_od$$

where *P*_dp-jt*_od*_ and *P*_dp-ft*_od*_ are the annual probabilities of domestic pigs’ exposure to ASFV per origin territory per prefecture caused by foreign job trainees and foreign tourists, respectively.

### 4.18. Semi-Quantitative Model to Assess the Potential Exposure of Wild Boar to ASFV

The semi-quantitative stochastic model was built to assess the risk of ASFV exposure to wild boars in the 47 prefectures of Japan arising from PPAP. The present approach was similar to the one used to assess the risk of ASF introduction into the EU [3,12,13,16]. To compare the risk of transmission from each origin territory to each of the 47 prefectures of Japan, five risk estimators were assigned to set the final index: (1) origin territory risk, (2) tourist-related risk, (3) wild boar distribution, (4) probability of wild boar habitat, and (5) probability of direct or indirect contact between tourists and wild boars. These five risk estimators were divided into ranks, and all five ranked scores were subsequently combined to obtain the relative risk value for each prefecture from each territory, as risk depends on the simultaneous occurrence of these estimators. The semi-quantitative model was constructed in Microsoft Excel (Microsoft, Redmond, WA, USA). In each parameter, from territory to destination prefecture, a score from 1 to 5 was assigned based on natural breaks in the data adjusted by the Jenks natural breaks classification method [40] using the Real Statistics Resource Pack [41]. The overall risk (Rwb_od) was calculated semi-quantitatively using the following formula:

(8) Rwb_od=Rasf_o×Rvisit_od×Rprsc_d×Rarea_d×Rcontact_d

Details of the model inputs and references are presented in Table 8 and are described in the following paragraphs.

### 4.19. Origin Territory Risk (R_asf_o_)

In the present study, the origin territory risk (*R*_asf*_o*_) was regarded as equal to the probability of the PPAP being contaminated with ASFV in the given territory (*P*_asf*_o*_). The value of *P*_asf*_o*_ was calculated by the approach used above. The obtained value of *P*_asf*_o*_ was then ranked from 1 to 5 based on the Jenks natural breaks classification (*R*_asf*_o*_). 

### 4.20. Tourist-Related Risk (R_visit_od_)

In the present study, tourists visiting Japan for sightseeing purposes (*N*_sight_) were assumed to carry the potential risk of exposing wild boar to ASFV via PPAP. Tourists may come into contact with wild boars in the natural areas of wild boar inhabit. First, the annual number of tourists coming to Japan for sightseeing purposes per origin territory per destination prefecture (*N*_sight*_od*_) was calculated using a similar approach to that already mentioned above. The value of *N*_sight*_od*_ was calculated using the following formula:

(9) Nsight_od=Nall_od×(Nqsight_od/Nqall_od)

where *N*_qsight*_od*_ is the number of tourists coming to Japan per territory per destination prefecture during the period of the survey. 

The mean proportion of tourists visiting natural areas per territory (*W*_nature_*o*_) was estimated based on a survey by the Japan Tourism Agency [42]. The annual number of tourists visiting natural areas per territory per destination prefecture (*N*_nature_*od*_) was estimated by multiplying the value of *N*_sight*_od*_ by the value of *W*_nature_*o*_. The annual weight of PPAP arriving in the natural area in each destination prefecture from each origin territory (*V*_nature*_od*_) was calculated by multiplying the value of *N*_nature_*od*_ by the value of *V*_enter*_vo*_, which had already been estimated above. The obtained value of *V*_nature*_od*_ was then ranked from 1 to 5 based on Jenks natural breaks (*R*_visit*_od*_).

### 4.21. Wild Boar Distribution (R_prsc_d_)

The wild boar distribution per destination prefecture (*R*_prsc*_d*_) was estimated based on two risk sub-estimators: the proportion of 5 km^2^ meshes with a wild boar presence per prefecture (*W*_prsc*_d*_) and the density of captured wild boar per wild boar presence mesh per prefecture (*D*_capt*_d*_). The annual number of captured wild boar per prefecture (*N*_capt*_d*_) was used for the risk calculation since no estimation of the wild boar population had been conducted at the prefecture level in Japan. The value of *W*_prsc*_d*_ was calculated by dividing the number of 5 km^2^ meshes with a wild boar presence per prefecture (*N*_mesh-wb*_d*_) by the total number of 5 km^2^ meshes per prefecture (*N*_mesh-all*_d*_). The information on the number of 5 km^2^ meshes with a wild boar presence was obtained from a survey by the Biodiversity Center of Japan, Ministry of the Environment [43]. The value of *D*_capt*_d*_ was calculated by dividing the annual number of captured wild boar per prefecture (*N*_capt*_d*_) by the number of 5km^2^ meshes with wild boar presence per prefecture (*N*_mesh-wb*_d*_ ). The value of *N*_capt*_d*_ was obtained from the Ministry of Environment statistics [44]. The values obtained for *W*_prsc*_d*_ and *D*_capt*_d*_ were converted to an adjusted risk rank on a scale of 1 to 5 using Jenks natural breaks. The value of *R*_prsc*_d*_ was then calculated as the product of the values of *W*_prsc*_d*_ and *D*_capt*_d*_.

### 4.22. Probability of Wild Boar Suitable Habitat (R_area_d_)

The probability that there would be suitable habitat for wild boar in each prefecture (*R*_area*_d*_) was estimated based on the proportion of the wild boar suitable habitat area [Quality of available habitat (QAH) levels of > 1.5] in the total area of each prefecture (*W*_habit*_d*_). The QAH map is a cartographic tool previously suggested as a potential tool for managing ASF [45]. Briefly, it is a standardised distribution map based on GLOBCOVER that quantifies the QAH for wild boar. The QAH map provided seven levels of QAH, namely 0, ‘absent’; 0.1, ‘unsuitable’; 0.5, ‘worst suitable area’; 1, ‘suitable areas for food or shelter’; 1.5, ‘suitable areas for food and shelter, but used mainly for one or the other’; 1.75, ‘suitable areas for food and shelter, but mainly used for food’; and 2, ‘suitable areas for both food and shelter’. The value of *W*_habit*_d*_ was calculated by dividing the area of the wild boar suitable habitat (*A*_suit-habit*_d*_) by the total area of the prefecture (km^2^) (*A*_all*_d*_) obtained from the Geospatial Information Authority of Japan database [46]. The obtained value was converted to an adjusted risk rank on a scale of 1 to 5 using Jenks natural breaks (*R*_area*_d*_).

### 4.23. Risk of Direct or Indirect Contact between Tourists and Wild Boars (R_contact_d_)

The probability of direct or indirect contact between tourists and wild boar per prefecture (*R*_contact*_d*_) was calculated based on seven risk sub-estimators: the proportion of the area with QAH levels of 1 in the total area of the prefecture (*W*_qah1*_d*_); the number of bus stops, roadside rest area, train stations, and tourist resource points overlapping with the wild boar habitat area per square kilometre per prefecture (*N*_bus-over*_d*_, *N*_road-over*_d*_, *N*_train-over*_d*_, and *N*_tores-over*_d*_, respectively); the proportion of the prefecture covered by natural parks (*W*_npark*_d*_); and the number of hunters per prefecture (*N*_hunt*_d*_). According to Bosch et al., an area with QAH level of 1 with two km buffer area is the place where frequent contact between domestic pig and wild boar is likely to occur, suggesting that, due to the frequent emergence of wild boar in areas of human habitation, the risk of contact between tourists and wild boar should be at a certain level. The value of *W*_qah1*_d*_ was calculated by dividing the area with QAH levels of 1 (km^2^) (*A*_qah1*_d*_) by the value of *A*_all*_d*_. The value of *A*_qah1*_d*_ was calculated based on the QAH map as applied above. 

The values of *N*_bus-over*_d*_, *N*_road-over*_d*_, *N*_train-over*_d*_, and *N*_tores-over*_d*_ were included as risk sub-estimators since it was expected that an increase in the number of tourists could increase the risk of contact between tourists and wild boar. First, the number of bus stops per prefecture (*N*_bus*_d*_) was obtained from the MLIT database [48]. The obtained data was divided by the habitable area for wild boar per prefecture (km^2^) (*A*_habit*_d*_) to calculate the value of *N*_bus-over*_d*_ in ArcGIS 10.6.1 software (ESRI Inc., Redlands, CA, USA). The same method was applied to calculate the value of *N*_road-over*_d*_, *N*_train-over*_d*_, and *N*_tores-over*_d*_.

Since natural parks are intended not only to protect the landscape but also to contribute to maintaining biodiversity, a higher risk of contact between tourists and wild boar was estimated for these areas. The value of *W*_npark*_d*_ was calculated by dividing the area of the natural park per prefecture (km^2^) (*A*_nature*_d*_) by the value of *A*_all*_d*_. The information on the area of the natural park per prefecture (km^2^) was obtained from the MLIT database [48].

The number of hunters per prefecture (*N*_hunt*_d*_) was also included as one of the risk sub-estimators since hunters should be familiar with wild animal habitats and have the potential to lead tourists to areas inhabited by wild boar. The information on the number of hunters was obtained from the Ministry of Environment statistics [42].

Each of the obtained risk sub-estimators was converted to an adjusted risk rank on a scale of 1 to 5 using Jenks natural breaks. The value of *R*_contact*_d*_ was calculated as the product of seven risk sub-estimators, and then it was ranked from 1 to 5 based on Jenks natural breaks again.

### 4.24. Sensitivity Analysis

In the model of ASFV exposure to domestic pigs, to identify the most influential parameters to model outputs among the input parameters in the present model, Spearman correlation coefficients (ρ*_i_*) were calculated between each input and the annual probability of ASFV exposure to domestic pigs in Japan through two considerable pathways. Inputs with ρ*_i_*≥ 0.4 that contributed ≥ 10% to the variance of the output were identified as the most influential parameters in the model and were analysed in detail using the advanced sensitivity analysis tool in @RISK 7.6, with 10,000 iterations for each scenario. A total of 10 scenarios were assessed for each selected parameter by changing the base values in 10 consecutive steps from a minimum of a 50% reduction to a maximum of a 50% increase. 

In the model of ASFV exposure to wild boar, the approaches of Torre et al. and Bosch et al. [3,49] were followed—a jackknife sensitivity analysis was conducted in Microsoft Excel that systematically left out each risk estimator from the model to identify the most influential estimators [50]. 

## Figures and Tables

**Table 1:** Mean annual risk of African Swine Fever Virus (ASFV) exposure to domestic pigs in prefectures of Japan via pork products brought in air passenger’s luggage (PPAP) by foreign job trainees per origin territory.

Territory of Origin	Risk Value [95% Confidence Interval]
Vietnam	0.168 [0.000–0.595]
the Philippines	0.002 [0.000–0.010]
Cambodia	1.2 × 10^−4^ [0.000–8.1 × 10^−4^]
China	3.8 × 10^−5^ [0.000–2.1 × 10^−4^]
Mongolia	4.0 × 10^−6^ [0.000–1.7 × 10^−5^]
Myanmar	1.6 × 10^−6^ [0.000–9.2 × 10^−6^]
Thailand	1.6 × 10^−7^ [0.000–8.7 × 10^−7^]
Sri Lanka	9.9 × 10^−11^ [0.000–0.000]

**Table 2:** The risk result of ASFV exposure to domestic pigs in prefectures of Japan via PPAP by foreign job trainees per prefecture.

Destination Prefecture	Risk Value [95% Confidence Interval]
Chiba	0.026 [0.000–0.093]
Ibaraki	0.020 [0.000–0.075]
Saitama	0.017 [0.000–0.062]
Miyazaki	0.013 [0.000–0.050]
Iwate	0.012 [0.000–0.047]
Aichi	0.012 [0.000–0.046]
Gunma	0.011 [0.000–0.044]
Hokkaido	0.010 [0.000–0.044]
Kanagawa	0.010 [0.000–0.039]
Ehime	0.009 [0.000–0.037]

**Table 3:** Mean annual risk of ASFV exposure to domestic pigs in prefectures of Japan via PPAP by foreign tourists per origin territory.

Territory of Origin	Risk Value [95% Confidence Interval]
Vietnam	0.042 [0.000–0.179]
the Philippines	0.007 [0.000–0.026]
China	0.001 [0.000–0.005]
the Russian Federation	3.0 × 10^−4^ [0.000–5.0 x 10^−4^]
Cambodia	2.9 × 10^−4^ [0.000–6.2 × 10^−4^]
South Korea	1.6 × 10^−4^ [0.000–6.4 × 10^−4^]
Poland	8.3 × 10^−6^ [0.000–0.000]
Myanmar	2.9 × 10^−6^ [0.000–2.5 × 10^−6^]
Germany	7.3 × 10^−7^ [0.000–2.0 × 10^−6^]
Italy	3.6 × 10^−7^ [0.000–2.5 × 10^−7^]

**Table 4:** Mean annual risk of ASFV exposure to domestic pigs in prefectures of Japan via PPAP by foreign tourists per prefecture.

Destination Prefecture	Risk Value [95% Confidence Interval]
Chiba	0.012 [0.000–0.053]
Aichi	0.010 [0.000–0.041]
Nagasaki	0.005 [0.000–0.019]
Oita	0.005 [0.000–0.017]
Hokkaido	0.004 [0.000–0.014]
Yamanashi	0.004 [0.000–0.013]
Mie	0.002 [0.000–0.007]
Miyazaki	0.002 [0.000–0.007]
Ishikawa	0.002 [0.000–0.006]
Saitama	0.001 [0.000–0.003]

**Table 5:** Mean annual risk of ASFV exposure to wild boar via PPAP per origin territory.

Territory of Origin	Jenks Score
Vietnam	5
Cambodia	4
the Philippines	4
the Russian Federation	4
China	3
Ethiopia	3
South Korea	3

**Table 6:** Description and parameterisation of model inputs for estimation of the risk of ASFV exposure to domestic pigs in prefectures of Japan.

Notation	Definition	Parameterisation	Values	Reference/Source
*V*_enter*_vo*_	Weight of PPAP (kg) being missed at quarantine per visitor per origin territory		Vmiss_o/Nall_o	
*V*_miss*_o*_	Annual weight of PPAP (kg) being missed at quarantine per origin territory		Vconf_o×Pmiss/(1−Pmiss)	
*N*_all*_o*_	Annual number of visitors coming to Japan per origin territory	Normal (µ, ⍺)	Nqall_o×(Nall/Nqall)	
*N*_all_	Annual number of visitors coming to Japan			[28]
*N*_qall_	Total number of tourists come to Japan during the period of survey			[29]
*N*_qall_*o*_	Total number of tourists come to Japan per origin territory during the period of survey			[29]
*P*_miss_	Probability of PPAP not detected at border controls in Japan	Triang (min, most likely, max)	Triang (0.2,0.5,0.9)	[14]
*V*_conf*_o*_	Weight of PPAP (kg) confiscated at quarantine per origin territory		Nconf_o×Vppap	
*N*_conf*_o*_	Annual number of PPAP confiscated at quarantine per origin territory			[25]
*V*_ppap_	Weight of PPAP (kg) per item	Triang (min, most likely, max)	Triang (0.04, 1.1, 9.7)	[11]
*N*_jt*_od*_	Adjusted annual number of foreign job trainees working on pig farms per origin territory per prefecture		(Nfjt_d/Nqfjt_d)×Nqj_o	
*N*_qjt*_od*_	Number of foreign job trainees per origin territory per prefecture in JPPA report in 2017			[23]
*N*_qjt*_d*_	Number of foreign job trainees per prefecture in JPPA report in 2017			[23]
*W*_fjt*_d*_	Proportion of farms accepting foreign job trainees per prefecture		Nqfjt_d/Nqf_d	
*W*_fjt*_r*_	Proportion of farms accepting foreign job trainees per region		Nqfjt_r/Nqf_r	
*N*_qfjt*_d*_	Number of farms accepting foreign job trainees per prefecture in the JPPA report in 2017			[23]
*N*_qf*_d*_	Total number of farms participating in the survey per prefecture in the JPPA report in 2017			[23]
*N*_qfjt*_r*_	Number of farms accepting foreign job trainees per region in the JPPA report in 2017			[23]
*N*_qf*_r*_	Total number of farms participating in the survey per region in the JPPA report in 2017			[23]
Mod-*W*_fjt*_d*_	Modified proportion of farms accepting foreign job trainees in prefecture d	Uniform (min, max)	min, max = Between *W*_fjt*_d*_ and *W*_fjt*_r*_	
*N*_fjt*_d*_	Estimated number of farms accepting foreign job trainees per prefecture		Mod−Wfjt_d×Nf_d	
*N*_f*_d*_	Number of pig farms per prefecture			[30]
*V*_jt*_od*_	Annual weight of PPAP (kg) brought into Japan by foreign job trainees per origin territory per destination prefecture		Njt_od×Venter_vo	
*W*_left_	Average proportion of meat leftover per family		0.03	[31]
*N*_vis_	Number of days foreign job trainees visit pig farm		313	[32]
*P*_ent*_od*_	Potential risk of leftover PPAP reach to pig farms		Pbs_d×Pbhv_o	
*P*_bs*_d*_	Level of farm biosecurity per prefecture	Triang (min, most likely, max)	Different values among prefectures	[33]
*W*_eco_	Proportion of farms introducing eco-feed activity			[23]
*I*_life*_o*_	Quality of life index			[34]
*P*_bhv*_o*_	Potential risk of worker’s behaviour per origin territory		(Ilife_Japan / Ilife_o)×Weco	
*V*_jexp*_od*_	The amount of cumulative exposure of PPAP (kg) to domestic pigs by foreign job trainees		$V{\mathrm{jt}}_{od}\times W\mathrm{left}\times N\mathrm{vis}\times P\mathrm{ent}\_od$	
*P_dp-jt_od_*	Annual probability of domestic pigs’ exposure to ASFV in prefecture d caused by foreign job trainees from territory o		$1-{\left(1-P_{asf\_o}\right)}^{V_{jexp\_od}}$	
*P*_asf*_o*_	Probability of 1 kg of PPAP being contaminated with ASFV in origin territory			[10,14]
*N* *_qfrd_od_*	The number of tourists coming to Japan to see their friends or family per territory per destination prefecture during the period of survey			[29]
*N*_frd*_od*_	Annual number of tourists coming to Japan to see their friends or family per origin territory per destination prefecture	Normal (µ, ⍺)	Nall_od×(Nqfrd_od/Nqall_od)	
*N*_qall_*od*_	Total number of tourists coming to Japan per origin territory per destination prefecture during the period of survey			[29]
*N*_all_*od*_	Annual number of tourists per origin territory per destination prefecture	Normal (µ, ⍺)	Nqall_od×(Nall/Nqall)	
*P*_pass*_d*_	Probability of tourists delivering PPAP to pig farmers		(Npf_d×Nfami)/Npop_d	
*N*_pf_*d*_	Number of pig farmers per prefecture		Nemp_s×Nf_sd	
*N*_pop_*d*_	Total population per prefecture			[35]
*N*_emp_*s*_	Number of employees per farm size	Uniform (min, max)		[33]
*N*_f_*sd*_	Number of pig farms per farm size per prefecture			[30]
*N*_fami_	Average number of family members in Japan		2.47	[36]
*N*_pass*_od*_	Annual number of tourists delivering PPAP to pig farmers		Nfrd_od×Ppass_d	
*V*_pass*_od*_	Annual weight of PPAP (kg) delivering to pig farmers		Npass_od×Venter_vo	
*V*_fexp*_od*_	The amount of cumulative exposure of PPAP (kg) to domestic pigs by foreign tourists		Vpass_od×Wleft×Nvis×Pbs_d	
*P_dp-ft_od_*	Annual probability of domestic pigs’ exposure to ASFV in prefecture d caused by foreign tourists from territory o		$1-{\left(1-P_{asf{\_}_{o}}\right)}^{V_{fexp\_0d}}$	

**Table 7:** The expert opinion ranges of the importance of farm biosecurity for ASFV.

Description	Median	Mean	SD	CV
**Section 1: Entrance and exit of farm visitor**				
Set up a signboard to indicate its restriction area	3.5	3.7	1.11	0.30
Shower in / Shower out	5	4.3	0.94	0.22
Exchange clothes for farm	5	4.8	0.60	0.13
Exchange shoes for farm	5	4.8	0.60	0.13
Step-in disinfection tank for farm	4	3.8	0.99	0.26
Exchange clothes for pigpen	3.5	3.8	1.07	0.28
Exchange shoes for pigpen	4.5	4.0	1.15	0.29
Step-in disinfection tank for pigpen	3	3.4	1.11	0.33
Record of visitor’s information	4.5	3.8	1.53	0.41
Movement restriction of the human who visit overseas	5	4.7	0.62	0.13
**Section 2: Quarantine for pig introduction**				
Quarantine at isolation facility during a certain period	5	3.5	0.87	0.25
**Section 3: Manual for Introduction of materials**				
Materials are stored in a warehouse for a certain period and disinfected before introduction	5	3.5	1.22	0.35
**Section 4: Entrance and exit of the vehicles**				
Disinfection of vehicles before entrance	5	4.9	0.28	0.06
Appropriate disinfection of vehicles for pig transportation	5	5.0	0.00	0.00
Set the gate for disinfection	5	4.2	1.14	0.27
Set the power sprayer for disinfection	5	4.6	0.76	0.17
Dissemination of sprinkle lime for disinfection	4	3.8	0.83	0.22
Frequent check of the appropriateness of disinfection	5	4.8	0.43	0.09
**Section 5: Prevention for wild animal intrusion**				
Appropriate maintenance of pig farm	5	4.5	0.76	0.17
The setting of wire-mesh fence	5	4.9	0.28	0.06
Invasion prevention for wild birds	5	4.8	0.43	0.09
Invasion prevention for small animals	5	4.4	0.86	0.20

SD, standard deviation; CV, coefficient of variation.

**Table 8:** Description and parameterisation of model inputs for estimation of the risk of ASFV exposure to wild boars (WB) in the prefectures of Japan.

Notation	Definition	Parameterization	Values	Reference/Source
*R*_asf*_o*_	Risk of origin territory	Jenks natural breaks (1–5)	*P*_asf*_o*_	
*R*_visit*_od*_	Tourists related risk per origin territory per destination prefecture	Jenks natural breaks (1–5)	*V*_nature*_od*_	
*N*_sight_*od*_	Annual number of tourists coming to Japan for sightseeing per origin territory per destination prefecture		Nall_od×(Nqsight_od/Nall_od)	
*N*_qsight*_od*_	Number of tourists coming to Japan for sightseeing per origin territory per destination prefecture during the period of the survey			[29]
*W*_nature_*o*_	Proportion of tourists visiting natural area per origin territory	Mean		[42]
*N*_nature_*od*_	Annual number of tourists visiting natural area per origin territory per destination prefecture		Nsight_od×Wnature_o	
*V*_nature*_od*_	Annual weight of PPAP (kg) arrived at the natural area in the destination prefecture from origin territory		Nnature_od×Venter_vo	
*R*_prsc*_d*_	WB distribution per prefecture	Jenks natural breaks (1–5)	Wprsc_d×Dcapt_d	
*W*_prsc*_d*_	Proportion of mesh with WB presence per prefecture	Jenks natural breaks (1–5)	Nmesh−wb_d/Nmesh−all_d	
*N*_mesh-wb*_d*_	Number of 5km^2^ meshes with WB presence per prefecture			[43]
*N*_mesh-all*_d*_	Total number of 5 km^2^ meshes per prefecture			[43]
*D*_capt*_d*_	Density of captured WB per WB presence mesh per prefecture	Jenks natural breaks (1–5)	Ncapt_d/Nmesh−wb_d	
*N*_capt*_d*_	Annual number of captured WB per prefecture	Mean		[44]
*A*_habit*_d*_	Area for WB habitat per prefecture (km^2^)			[45]
*R*_area*_d*_	Probability of WB suitable habitat per prefecture	Jenks natural breaks (1–5)	Whabit_d	
*W*_habit*_d*_	Proportion of WB suitable habitat area in the total area of the prefecture		Asuit−habit_d×Aall_d	
*A*_suit-habit*_d*_	Area for wild boar suitable habitat per prefecture (km^2^)			[45]
*A*_all*_d*_	Total area of the prefecture (km^2^)			[46]
*R*_contact*_d*_	Probability of direct or indirect contact between tourists and WB per prefecture	Jenks natural breaks (1–5)	Wqah1_d×Nbus−over_d×Nroad−over_d×Ntrain−over_d×Ntores−over_d * Wnpark_d×Nhunt_d	
*W*_qah1*_d*_	Proportion of area with QAH levels of 1 in the total area of the prefecture	Jenks natural breaks (1–5)	Aqah1_d/Aall_d	
*A*_qah1*_d*_	Area with QAH levels of 1 (km^2^) per prefecture			[45]
*N*_bus*_d*_	Number of bus stops per prefecture			[47]
*N*_bus-over*_d*_	Number of bus stops overlapped with WB habitat area per square kilometer per prefecture	Jenks natural breaks (1–5)	Nbus_d/Ahabit_d	[45,47]
*N*_road*_d*_	Number of roadside rest area per prefecture			[47]
*N*_road-over*_d*_	Number of roadside rest area overlapped with WB habitat area per square kilometer per prefecture	Jenks natural breaks (1–5)	Nroad_d/Ahabit_d	[45,47]
*N*_train*_d*_	Number of train stations per prefecture			[47]
*N*_train-over*_d*_	Number of train stations overlapped with WB habitat area per square kilometer per prefecture	Jenks natural breaks (1–5)	Ntrain_d/Ahabit_d	[45,47]
*N*_tores*_d*_	Number of tourist resource points per prefecture			[47]
*N*_tores-over*_d*_	Number of tourist resource points overlapped with WB habitat area per square kilometer per prefecture	Jenks natural breaks (1–5)	Ntores_d/Ahabit_d	[45,47]
*W*_npark*_d*_	Proportion of natural park area in the prefecture	Jenks natural breaks (1–5)	Anature_d/Aall_d	
*A*_nature*_d*_	Area of natural park per prefecture (km^2^)			[47]
*N*_hunt*_d*_	Number of hunters per prefecture	Jenks natural breaks (1–5)		[44]
